# Identification and interpretation of TET2 noncanonical splicing site intronic variants in myeloid neoplasm patients

**DOI:** 10.1002/jha2.744

**Published:** 2023-06-21

**Authors:** Riku Das, Zheng Jin Tu, David S. Bosler, Yu‐Wei Cheng

**Affiliations:** ^1^ Department of Laboratory Medicine, Robert J. Tomsich Pathology and Laboratory Medicine Institute Cleveland Clinic Cleveland Ohio USA

**Keywords:** in silico prediction, myeloid neoplasm, next‐generation sequencing (NGS), noncanonical splicing site, ten‐eleven translocation 2, TET2, tumor suppressor

## Abstract

**Background**: DNA hypermethylation and instability due to inactivation mutations in Ten–eleven translocation 2 (*TET2*) is a key biomarker of hematological malignancies. This study aims at characterizing two intronic noncanonical splice‐site variants, c.3954+5_3954+8delGTTT and c.3954+5G>A.

**Methods**: We used *in silico* prediction tools, reverse transcription (RT)‐PCR, and Sanger sequencing on blood/bone marrow‐derived RNA specimens to determine the aberrant splicing.

**Results**: *In silico* prediction of both variants exhibited reduced splicing strength at the *TET2* intron 7 splicing donor site. RT‐PCR and Sanger sequencing identified a 62‐bp deletion at the exon 7, producing a frameshift mutation, p.Cys1298*.

**Conclusion**: This study provides functional evidence for two intronic *TET2* variants that cause alternative splicing and frameshift mutation.

## 1 INTRODUCTION

Ten‐eleven translocation (*TET*) is a three‐member family of genes, located on chromosome 4q24, that encodes for enzymes Tet methylcytosine deoxygenate. *TET2* are key epigenetic modifiers and tumor suppressor genes, crucial in hematological malignancies as well as normal hematopoiesis, immune cell activation, and clonal expansion [1, 2, 3]. *TET2* loss of function and deletion leading to the hypermethylated state of the genome is frequently detected in myeloid and lymphoid malignancies and clonal hematopoiesis of indeterminate potential. In high throughput genome‐wide studies, somatic deletion and loss of function *TET2* mutations are detected in 7–23% of acute myeloid leukemia (AML), 10%–20% of myelodysplastic syndrome (MDS)/myeloproliferative neoplasms (MPN), and 40%–50% chronic myelogenous leukemia patients [2, 4–6]. *TET2* regulates gene expression primarily through the demethylation of DNA 5‐methylcytosine (5mC) to 5‐hydroxymethyl cytosine (5hmC), requiring Fe^2+^ and α‐ketoglutarate (α‐KG) for their activity. Subsequently, *TET2* converts 5hmC to 5‐formylcytosine (5fC) and 5‐carboxy‐cytosine (5caC), facilitating the removal of 5fC and 5caC through the base‐excision repair pathway and playing a key role in DNA demethylation and transcription regulation [7]. *TET2* deoxygenase domain resides in its exons 4 through 11, which consists of a cysteine‐rich domain, a double‐stranded β‐helix (DSBH) domain, and a low‐complexity linker region [4, 8]. The DSBH domain harbors binding sites for Fe(II) and α‐ketoglutarate that help *TET2* binding to carry out the 5mC oxidation process.

The majority of inactivating or loss‐of‐function *TET2* variants are exonic and located throughout the coding exons, including 76.7% truncating, 17.4% missense and 4.8% splice sites as reported in the cBioportal database (on 02/05/2023). However, the lack of study in *TET2* noncanonical splicing site intronic variants has made it very challenging to interpret these variants for clinical usage. In fact, most clinical laboratories will either choose not to report these variants or classify them as unknown clinical significance. In a combination of in silico prediction, RT‐PCR, and Sanger sequencing analysis here we show the impact of two noncanonical splicing site intronic variants of *TET2*, a deletion c.3954+5_3954+8delGTTT and a recurrent substitution c.3954+5G>A, in producing frameshift mutations that expected to yield truncated, dysfunctional *TET2* gene products.

## 2 MATERIALS AND METHODS

### 2.1 Patient samples, DNA and RNA extraction, next‐generation sequencing

Genomic DNA and total RNA were extracted from either bone marrow or peripheral blood of the same specimens. A nonleukemic patient was used as the negative control. Two *TET2* intronic variants c.3954+5_3954+8delGTTT (present in one patient) and c.3954+5G>A (present in two patients) were identified from a 63‐gene hematologic neoplasm next‐generation sequencing (NGS) panel. For a given sample, the NGS minimum coverage requirement of targeted regions was >100X. Variants with variant allele fractions (VAFs) as low as 4% were identified. The study was conducted according to the approved protocols of Cleveland Clinic's Institutional Review Board (17–177 and 19–329).

### 2.2 *In silico* splicing prediction, RT‐PCR and Sanger sequencing

*In silico* splice prediction tools, including SpliceSiteFinder‐like, MaxEntScan, NNSplice, and GeneSplicer, integrated into the Alamut Visual Plus (Version v1.3, SOPHiA GENETICS), were used in this study [5]. PCR was performed using a forward primer specific to *TET2* Exon 6, 5′‐CCGAGACGCTGAGGAAATAC−3′ and a reverse primer specific to *TET2* Exon 8, 5′‐TGGACAGGTTTTGCAAATGA−3′. Sanger sequencing was conducted using BigDye Terminator 1.1 kits.

## 3 RESULTS

### 3.1 Identification of two intronic *TET2* variants in hematoneoplasm patients

**FIGURE 1:**
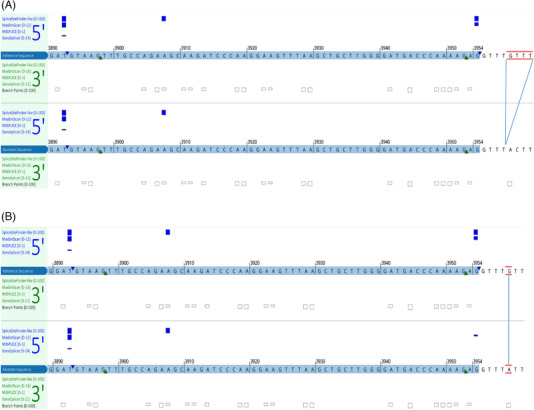
(A and B) *In silico* splicing prediction of *TET2* intronic variants of (A) c.3954+5_3954+8del, and (B) c.3954+5G>A. Variants are indicated by red lines. Four splicing predictors were used; the impact on gene splicing is indicated with vertical blue bars. The predicted strength of canonical splice donor signals at the splicing junctions is reduced in both variants compared to the wild‐type reference transcript. The gains of splicing signals were observed at an internal cryptic splice donor site.

Two novel *TET2* (NM_001127208.2) intron 7 variants with high VAFs were identified in three patients (Table 1). Patient 1 was diagnosed with acute myeloid leukemia, carried 4‐bp deletion, c.3954+5_3954+8delGTTT, with 42.5% VAF. Patient 2 and patient 3 with chronic myelomonocytic leukemia carry the substitution mutation, c.3954+5G>A, with 77.31% and 97.68% VAFs, respectively. As these variants are located very near to the intron 7 canonical splice donor sequence (GT) and within the 5′ splice site (5′ss) consensus sequence [6], we conducted an in silico splicing analysis to predict the impact of splicing. Our analyses showed a drastic decline of splicing strength at the *TET2* intron 7 splicing donor site in both variants, c.3954+5_3954+8delGTTT and c.3954+5G>A, in compared with the *TET2* wild‐type transcript (Figure 1A,B).

**TABLE 1:** Clinical characteristics and variant information.

Characteristics	Patient 1	Patient 2	Patient 3
Tumor type	Acute myeloid leukemia	Chronic myelomonocytic leukemia	Acute myeloid leukemia (with a history of chronic myelomonocytic leukemia)
Specimen source	Peripheral blood	Bone marrow	Peripheral Blood
*TET2* intronic variants	c.3954+5_3954+8delGTTT	c.3954+5G>A	c.3954+5G>A
	VAF = 42.5%	VAF = 77.31%	VAF = 97.68%
	Depth = 647	Depth = 335	Depth = 689

### 3.2 Confirmation of impact in splicing in two novel *TET2* variants

**FIGURE 2:**
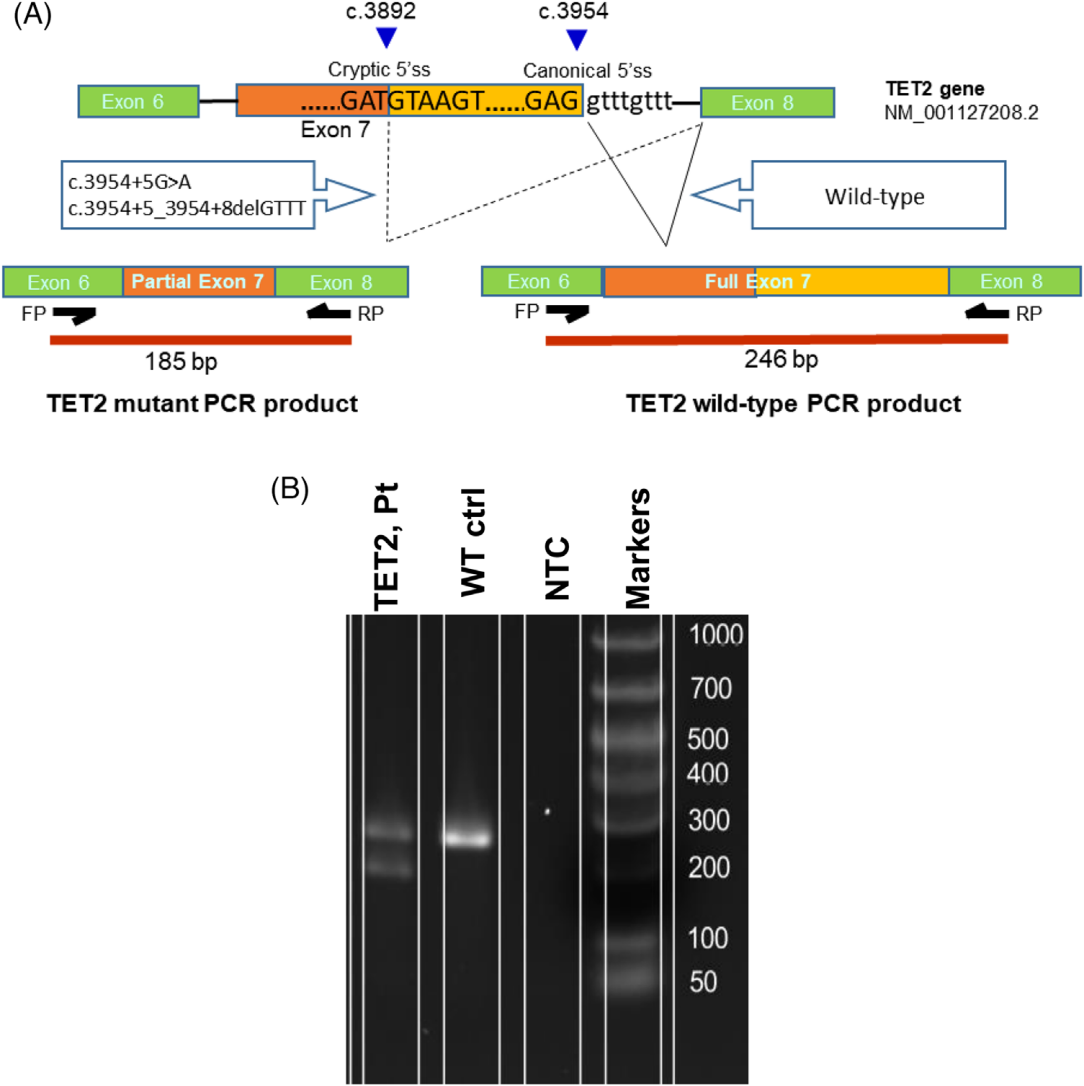
(A) Graphic representation showing *TET2* splicing products with or without *TET2* intronic variants (c.3954+5_3954+8delGTTT, c.3954+5G>A). Breakpoints at the cryptic and original 5′ splice site junctions are shown with blue triangles. The primer binding sites and subsequent PCR products in WT and mutant *TET2* spliced mRNA are also shown. (B) RT‐PCR analysis on RNA from a negative control patient and a patient with *TET2* c.3954+5_3954+8del variant. Gel electrophoresis image showing PCR products amplified using specific primers binding to Ex6 (forward) and Ex8 (reverse). Fragment size of 246 bp indicates *TET2* wild‐type sequences (ex6+ex7+ex8) and fragment of 185 bp size (ex6+ partial ex7+ex8) indicates the *TET2* splicing variant. Only wild‐type product was identified in the negative (WT) control. No visible product is shown in no template control (NTC).

**FIGURE 3:**
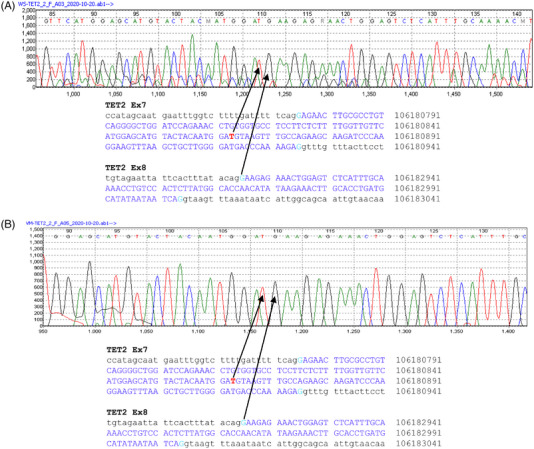
Sanger sequencing of the 185 bp size PCR products obtained from a polyacrylamide gel. Nucleotide sequences of wild‐type *TET2* transcript are shown at the bottom of each chromatogram. The coding exon sequences were highlighted in blue. The splicing junctions connecting exon 7 and exon 8, leading to partial depletion of exon 7, are indicated with black arrows. (A) Chromatogram of patient 1 fragment of *TET2* c.3954+5_3954+8del. (B) Chromatogram of patient 2 fragment of c.3954+5G>A substitution.

The two intronic variants’ impact on *TET2* gene splicing was further characterized by RT‐PCR and Sanger sequencing at the RNA level. As shown in Figure 2A,B, a band at approximately 246 bp region corresponding to the wild‐type sequence was seen in the negative (WT) control. However, for the patient with deletion mutation (patient 1), two bands were seen; one was at 246 bp, and the other appeared just below 200 bp. This ∼200 bp RT‐PCR product was also obtained for patients 2 and 3 (data not shown) along with 246 bp WT product. Sanger sequencing of the extracted 246 bp band from the WT control consists of all expected exonic sequences, including partial exon 6, full exon 7, and partial exon 8. Interestingly, sequencing of the ∼200 bp products from both cases of c.3954+5G>A and c.3954+5_3954+8delGTTT variants, as shown in Figure 3, revealed a 62‐bp deletion at the 3′ portion of the exon 7. This deletion used an exon 7 internal cryptic splicing site c.3892 (g.106180864) to produce an alternative splicing product that is predicted to cause a protein reading frameshift mutation, p.Cys1298*. These results demonstrate that both variants lead to the same premature termination codon that is expected to result in a truncating protein.

## 4 DISCUSSION

*TET2* is an epigenetic modifier; its mutations commonly occur alongside other driver mutations across AML, MPN, and MDS malignancies. Mutations of *TET2* are usually observed before the acquisition of other driver mutations contributing to genome instability and clonal evolution. Therefore, to understand the clonal structure, proliferative, and differentiation capacity of neoplastic cells in the hematologic neoplasm, and identification of loss‐of‐function variants in *TET2* is crucial. We have shown two intronic variants, c.3954+5_3954+8delGTTT and c.3954+5G>A, allow the splicing machinery to bypass the canonical 5′ splice donor site and activate a cryptic splicing sequence within exon 7, leading to exclusion of a portion of exon 7. This results in a frameshift mutation expected to produce a Tet2 truncating protein (p.Cys1298*) devoid of the majority of Tet2 oxygenase domain and the downstream amino acids. In addition, this study warrants the adoption of follow‐up confirmatory steps when *TET2* intronic variants are detected for accurate variant interpretation and classification.

The activation of 5′ cryptic or generation of *de novo* splice sites is mostly due to alteration in the first intron nucleotide (+1) or +5 position [9]. Position +5 in the intron is required for base pairing with U1 and U6 snRNA components of spliceosomes during the splicing process [10]. Since both variants, c.3954+5_3954+8delGTTT and c.3954+5G>A, in our study encompass the +5G intron position, our finding of the splicing product with a 62‐bp deletion of exon 7 corroborates with our understanding of pre‐mRNA splicing mechanism and demonstrates the usefulness of *in silico* prediction tools.

**FIGURE 4:**
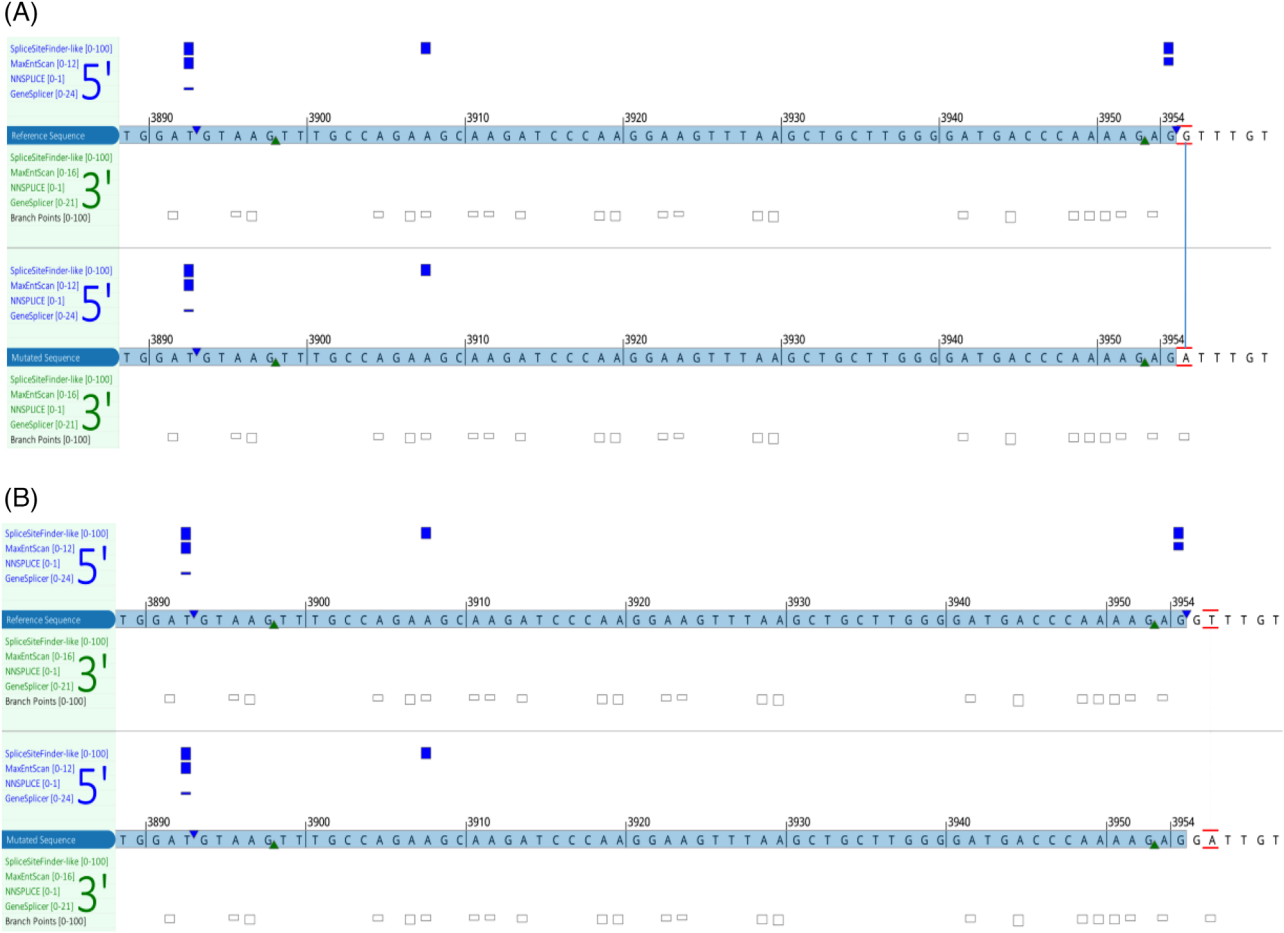
(A and B) Examples of in silico splicing prediction of *TET2* canonical splice donor variants of (A) c.3954+1G>A, and (B) c.3954+2T>A substitutions. Four splicing predictors were used; the impact on gene splicing is indicated with vertical blue bars. The predicted strength of canonical splice donor signals at the splicing junctions is reduced in both variants compared to the wild‐type reference transcript. The gains of splicing signals were observed at the same internal cryptic splice donor site as shown in Figure 1.

Sequence changes at the *TET2* intron 7 canonical splice donor site have been widely documented in the COSMIC database, including c.3954+1G>A (COSV54396751) [11, 12], c.3954+1G>T (COSV54397716) [13], c.3954+2T>G (COSV54429204), and c.3954+2T>A (COSV105020527) [14, 15]. Because *TET2* is a tumor suppressor gene, splicing site variants are predicted to cause gene inactivation and oncogenic events [13, 16]. In fact, shown in Figure 4, *in silico* predictions indicate that all the above‐mentioned COSMIC variants (positioned at c.3954+1 and c.3954+2) activate the identical exon 7 cryptic internal splicing site at c.3892 (g.106180864) shown in this study and are expected to produce the same truncating protein product, p.Cys1298*. The fact that the presented two *TET2* intronic variants and the exon 7 canonical splice donor site variants, all result in the same truncating gene product, highlights the importance of classifying both c.3954+5G>A and c.3954+5_3954+8delGTTT variants as potential clinical significance. It is worth mentioning that the c.3954+5G>A variant (rs575928986, dbSNP151) has a relatively rare minor allele frequency of 0.0032% (gnomAD v2.1) in the general population. However, in the disease cohort of our study, we have identified three such cases in a total of 2164 myeloid neoplasm patients, equating to a 0.139% instance rate. This increase of two orders of magnitude in mutation rate in the disease cohort and the evidence of producing a truncating protein strongly suggest that these two intronic variants are highly associated with the pathogenicity of myeloid neoplasms.

Due to a lack of functional evidence *TET2* intronic variants were not reported for the diagnosis and management of the patients’ disease. However, it is very likely that the *TET2* intronic variants contributed to the myeloid clonal evolution in these patients and provided a persistent mutational burden. These variants were detected at very high variant allele frequency (Table 1). Additionally, follow‐up sequencing for patients 2 and 3 detected the *TET2* intronic variants (c.3954+5G>A) at increased variant allele fractions with persistent disease phenotype. In addition to the *TET2* variants, all patients carried other known driver mutations in either *FLT3*, *KRAS, JAK2*, or *CBL* genes. Since the sequencing test was not performed at the early stages of disease manifestation, one cannot say for certain that *TET2* intronic variants alone acted as a founder mutation.

Altogether, this study identifies two *TET2* intronic variants that are potentially pathogenic. We present a cost‐effective approach, by combining the in silico prediction, RT‐PCR, and Sanger sequencing to determine the significance of potential splicing site variants. A clinical laboratory may adopt this approach to the standard operating procedure to improve the variant triage process.

## AUTHOR CONTRIBUTIONS

YWC designed the study. ZJT and DB collected data. DB provided clinical consultation and regulatory support for the cases. RD, ZJT, and YWC performed analyses. YWC supervised the study. RD and YWC drafted the manuscript. All authors approved the final manuscript.

## CONFLICT OF INTEREST STATEMENT

The authors declare that there is no conflict of interest that could be perceived as prejudicing the impartiality of the research reported.

## CLINICAL TRIAL REGISTRATION (INCLUDING TRIAL NUMBER)

This study has not been registered for any clinical trial.

## PATIENT CONSENT STATEMENT

n/a

## ETHICS STATEMENT

The study was conducted according to the approved protocols of Cleveland Clinic's Institutional Review Board (IRB; 17‐177 and 19‐329).

## Data Availability

Data are available upon request due to privacy/ethical restrictions.

## References

[1] Jiang S . Tet2 at the interface between cancer and immunity. Commun Biol. 2020;3(1):667. 33184433 10.1038/s42003-020-01391-5 PMC7661537

[2] Delhommeau F , Dupont S , Della Valle V , James C , Trannoy S , Masse A , et al. Mutation in TET2 in myeloid cancers. N Engl J Med. 2009;360(22):2289–301. 19474426 10.1056/NEJMoa0810069

[3] Kunimoto H , Nakajima H . TET2: a cornerstone in normal and malignant hematopoiesis. Cancer Sci. 2021;112(1):31–40. 33048426 10.1111/cas.14688 PMC7780023

[4] Bussaglia E , Anton R , Nomdedeu JF , Fuentes‐prior P. TET2 missense variants in human neoplasia. A proposal of structural and functional classification. Mol Genet Genomic Med. 2019;7(7):e00772. 31187595 10.1002/mgg3.772 PMC6625141

[5] Das R , Jakubowski MA , Spildener J , Cheng YW . Identification of novel MET exon 14 skipping variants in non‐small cell lung cancer patients: a prototype workflow involving in silico prediction and RT‐PCR. Cancers. 2022;14(19):4814. 36230737 10.3390/cancers14194814 PMC9563401

[6] Roca X , Sachidanandam R , Krainer AR . Intrinsic differences between authentic and cryptic 5' splice sites. Nucleic Acids Res. 2003;31(21):6321–33. 14576320 10.1093/nar/gkg830 PMC275472

[7] Ito S , Shen L , Dai Q , Wu SC , Collins LB , Swenberg JA , et al. Tet proteins can convert 5‐methylcytosine to 5‐formylcytosine and 5‐carboxylcytosine. Science. 2011;333(6047):1300–3. 21778364 10.1126/science.1210597 PMC3495246

[8] Hu L , Li Z , Cheng J , Rao Q , Gong W , Liu M , et al. Crystal structure of TET2‐DNA complex: insight into TET‐mediated 5mC oxidation. Cell. 2013;155(7):1545–55. 24315485 10.1016/j.cell.2013.11.020

[9] Buratti E , Chivers M , Kralovicova J , Romano M , Baralle M , Krainer AR , et al. Aberrant 5' splice sites in human disease genes: mutation pattern, nucleotide structure and comparison of computational tools that predict their utilization. Nucleic Acids Res. 2007;35(13):4250–63. 17576681 10.1093/nar/gkm402 PMC1934990

[10] Crispino JD , Sharp PA . A U6 snRNA:pre‐mRNA interaction can be rate‐limiting for U1‐independent splicing. Genes Dev. 1995;9(18):2314–23. 7557384 10.1101/gad.9.18.2314

[11] Sjoblom T , Jones S , Wood LD , Parsons DW , Lin J , Barber TD , et al. The consensus coding sequences of human breast and colorectal cancers. Science. 2006;314(5797):268–74. 16959974 10.1126/science.1133427

[12] Tefferi A , Lim KH , Abdel‐Wahab O , Lasho TL , Patel J , Patnaik MM , et al. Detection of mutant TET2 in myeloid malignancies other than myeloproliferative neoplasms: CMML, MDS, MDS/MPN and AML. Leukemia. 2009;23(7):1343–5. 19295549 10.1038/leu.2009.59 PMC4654626

[13] Papaemmanuil E , Gerstung M , Bullinger L , Gaidzik VI , Paschka P , Roberts ND , et al. Genomic classification and prognosis in acute myeloid leukemia. N Engl J Med. 2016;374(23):2209–21. 27276561 10.1056/NEJMoa1516192 PMC4979995

[14] Saint‐Martin C , Leroy G , Delhommeau F , Panelatti G , Dupont S , James C , et al. Analysis of the ten‐eleven translocation 2 (TET2) gene in familial myeloproliferative neoplasms. Blood. 2009;114(8):1628–32. 19564637 10.1182/blood-2009-01-197525

[15] Swierczek SI , Yoon D , Bellanne‐Chantelot C , Kim SJ , Saint‐Martin C , Delhommeau F , et al. Extent of hematopoietic involvement by TET2 mutations in JAK2V(6)(1)(7)F polycythemia vera. Haematologica. 2011;96(5):775–8. 21273266 10.3324/haematol.2010.029678 PMC3084927

[16] Bernard V , Gebauer N , Dinh T , Stegemann J , Feller AC , Merz H . Applicability of next‐generation sequencing to decalcified formalin‐fixed and paraffin‐embedded chronic myelomonocytic leukaemia samples. Int J Clin Exp Pathol. 2014;7(4):1667–76. 24817963 PMC4014247

